# Beyond clinical genomics: addressing critical gaps in One Health AMR surveillance

**DOI:** 10.3389/fmicb.2025.1596720

**Published:** 2025-04-28

**Authors:** Diana Vallejo-Espín, Jael Galarza-Mayorga, Liliana Lalaleo, William Calero-Cáceres

**Affiliations:** ^1^Biotechnology Program, Department of Food and Biotechnology Science and Engineering, Universidad Técnica de Ambato, Ambato, Ecuador; ^2^UTA-RAM-One Health, Group for Universal Advance in Bioscience, Department of Food and Biotechnology Science and Engineering, Universidad Técnica de Ambato, Ambato, Ecuador

**Keywords:** antimicrobial resistance, whole-genome sequencing, shotgun metagenomics, One Health, genomic surveillance

## Abstract

Antimicrobial resistance (AMR) poses an escalating global threat that demands comprehensive surveillance approaches beyond traditional clinical contexts. Although next-generation sequencing (NGS), particularly whole-genome sequencing (WGS), has revolutionized AMR surveillance, current implementation predominantly targets clinical isolates, largely neglecting critical environmental and animal reservoirs. Consequently, significant gaps persist in our understanding of AMR dynamics across diverse ecosystems. This Perspective emphasizes the urgent need to adopt an integrated genomic framework, combining isolate-based WGS with shotgun metagenomics within a cohesive One Health strategy. Such an integrated approach would significantly enhance the detection, tracking, and containment of resistance determinants, facilitating proactive rather than reactive AMR management. Achieving this vision requires global standardization of sequencing methods, harmonization of bioinformatics pipelines, and strengthened cross-sectoral collaboration to ensure timely interventions against AMR threats worldwide.

## 1 Introduction

The projection that antimicrobial resistance (AMR) could cause 10 million deaths annually by 2050, has reinforced its status as one of the greatest global health threats of the 21st century (Naghavi et al., 2024). Addressing AMR requires a multisectoral approach, particularly in understanding the evolution, reservoirs, and transmission pathways of resistant pathogens to implement effective containment strategies. However, the intricate nature of AMR drivers within various ecosystems impedes the comprehensive understanding of its dynamics. Next-generation sequencing (NGS) has transformed genomic surveillance, providing unparalleled insights into the evolution of antimicrobial resistance (Hu et al., 2024). However, its application is still fragmented, primarily confined to clinical environments like hospitals and national reference laboratories. This narrow focus does not encompass the wider resistome, overlooking essential environmental and animal reservoirs. This Perspective highlights the critical need for a cohesive AMR surveillance framework within a One Health context, integrating whole-genome sequencing (WGS) of clinical isolates with shotgun metagenomics, aiming to create a thorough, cross-sectoral approach for tracking and reducing the spread of resistance.

## 2 The role of genomics in AMR surveillance

### 2.1 Advances in NGS for AMR detection

NGS has transformed microbiological workflows, offering high-resolution characterization of AMR mechanisms in ways that were previously fragmented and labor-intensive. Traditionally, microbiologists relied on multiple complex techniques, including phenotypic assays, PCR-based resistance gene detection, multilocus sequence typing (MLST), pulsed-field gel electrophoresis (PFGE), and plasmid profiling, to identify resistance genes, virulence factors, and epidemiological relationships (Calero-Cáceres et al., 2023). These approaches, while informative, were time-consuming and often lacked the resolution needed to track AMR evolution and transmission in real time.

The advent of WGS has streamlined these processes, providing a single, comprehensive method for detecting antimicrobial resistance genes (ARGs), mobile genetic elements (MGEs), virulence traits, clonal relatedness, and phylogenetic profiles in one sequencing run. WGS is now a cornerstone of AMR surveillance in clinical settings, facilitating outbreak detection, strain characterization, and retrospective epidemiological investigations (Waddington et al., 2022). Meanwhile, shotgun metagenomics broadens the scope of AMR surveillance, allowing for the detection of resistance determinants directly from complex environmental and community samples, capturing AMR dynamics in unculturable bacteria and understudied reservoirs (Pillay et al., 2022). Together, these technologies are reshaping AMR monitoring across human, animal, and environmental health sectors, providing an unprecedented opportunity to trace resistance evolution and dissemination holistically.

### 2.2 Challenges in implementing genomic AMR surveillance

Despite significant advancements, genomics-based AMR surveillance remains fragmented, predominantly focused on clinical settings with insufficient integration across One Health compartments. WGS and metagenomics have yet to be fully incorporated into veterinary and environmental health programs, largely due to technical and financial barriers, including high sequencing costs, bioinformatics complexity, and the absence of standardized workflows for data analysis and resistance gene annotation (Calero-Cáceres et al., 2024). Additionally, inconsistencies in sequencing protocols across laboratories and countries hinder cross-sector comparability, limiting the ability to track resistance transmission across geographic and ecological boundaries (Ristori et al., 2024).

To maximize the impact of WGS and metagenomics in AMR surveillance, a globally coordinated and harmonized framework is needed. Standardizing sequencing methodologies, bioinformatics pipelines, and data-sharing platforms will facilitate interoperability across human, animal, and environmental health sectors (Calero-Cáceres et al., 2024). Addressing resource constraints in low- and middle-income countries (LMICs) through investments in capacity-building, real-time data integration, and public-private partnerships will be crucial to bridging existing gaps and establishing a truly comprehensive AMR monitoring system (Calero-Cáceres, 2024).

### 2.3 Expanding AMR surveillance beyond clinical settings

The global challenge of AMR extends beyond clinical settings. While hospital-based surveillance has provided critical insights into AMR evolution, its scope remains narrow, capturing only a fraction of the broader resistance landscape. The environmental and animal reservoirs of AMR—wastewater, agricultural runoff, food, wildlife, and airborne microbiota—are under-monitored, limiting our ability to track resistance dissemination effectively (Endale et al., 2023). Moreover, the lack of standardized sampling methodologies, sequencing approaches, and data-sharing frameworks creates gaps in surveillance, hindering global efforts to mitigate resistance spread (Black et al., 2020).

Expanding surveillance beyond hospitals requires a dual strategy: (i) WGS of bacterial isolates from clinical and animal settings and (ii) Shotgun metagenomics for complex matrices in environmental and community settings. While WGS provides high-resolution data on priority pathogens and emerging resistance mechanisms, metagenomics enables the identification of resistance hotspots and reservoirs of dissemination, both of which are critical to One Health AMR surveillance (Black et al., 2020).

## 3 Integrating whole-genome and metagenomic surveillance in a One Health framework

### 3.1 Isolate-based WGS for high-priority pathogens

**Figure 1:**
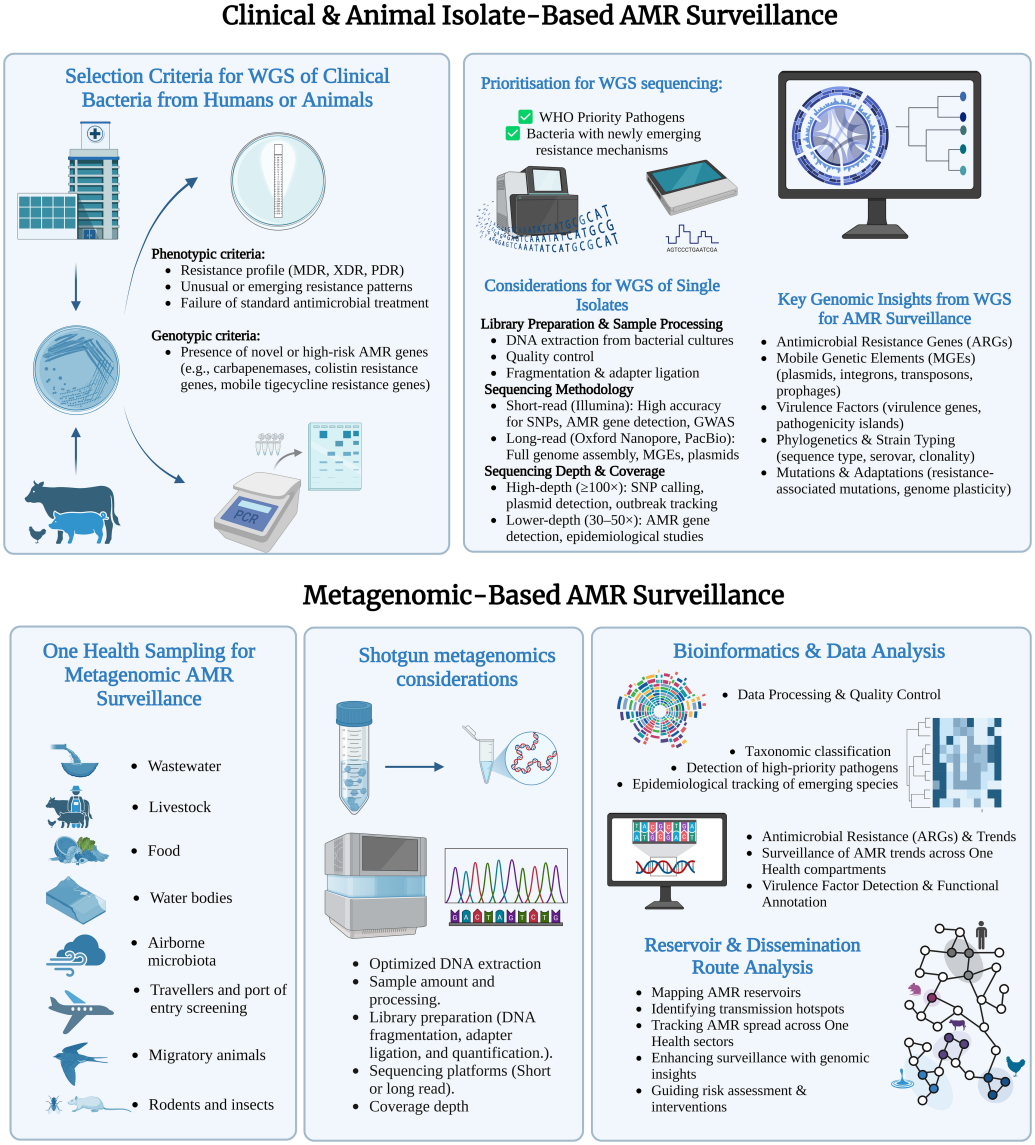
Integrative framework for genomic surveillance of antimicrobial resistance (AMR) combining isolate-based whole-genome sequencing (WGS) and shotgun metagenomics within a One Health approach. Figure was created by Biorender.

WGS is emerging as the most comprehensive approach for tracking antimicrobial-resistant bacteria in human and animal health, providing high-resolution detection of genetic resistance determinants, MGEs, and insights into strain evolution (Figure 1). Its implementation in AMR surveillance provides a robust framework for identifying high-risk strains, tracing transmission pathways, and monitoring the emergence of novel resistance mechanisms. Prioritizing WGS for WHO Priority Pathogens and newly emerging resistant bacteria is essential, particularly for strains exhibiting multidrug-resistant (MDR) or extensively drug-resistant (XDR) phenotypes (Calero-Cáceres et al., 2023; Almutairy, 2024; World Health Organization, 2024).

WGS enables precise characterization of genetic determinants associated with resistance dissemination, including plasmids, integrons, transposons, prophages, and mutations known to confer antimicrobial resistance (Algarni et al., 2022). Integrating phylogenetic analysis and strain typing improves our understanding of epidemiological linkages, delineates transmission routes, identifies reservoirs, and highlights bacterial populations facilitating resistance gene transfer. However, WGS alone cannot characterize unculturable microbial reservoirs, underscoring the complementary need for metagenomics to capture the broader resistome across complex ecosystems.

### 3.2 Metagenomic-based AMR surveillance

While WGS provides high-resolution insights into individual resistant bacteria, metagenomics enables a more comprehensive analysis of AMR within complex microbial communities, overcoming the limitations of culture-dependent methods (Pillay et al., 2022). By directly sequencing environmental DNA, metagenomics allows for the detection of resistance determinants in diverse reservoirs, including wastewater, food production systems, livestock environments, airborne microbiota, migratory animals, and vectors such as rodents and insects (Duarte et al., 2021). These compartments serve as critical hotspots for resistance gene exchange, driving the emergence and dissemination of novel resistance mechanisms across One Health sectors (Figure 1).

Beyond antimicrobial resistance gene (ARG) identification, metagenomic analysis facilitates the functional annotation of virulence factors, the epidemiological tracking of high-priority pathogens, and the surveillance of AMR trends at a broader ecological scale. Such data are essential for pinpointing transmission hotspots and assessing risks associated with environmental and community exposures, enabling early intervention before clinical outbreaks occur.

Integrating metagenomics with isolate-based WGS further strengthens surveillance efforts by providing complementary evidence that isolates alone cannot, supporting early detection of emerging resistance threats. Therefore, standardization of sequencing depth, bioinformatics pipelines, and data-sharing protocols across sectors will be essential for optimizing the effectiveness of metagenomics in AMR monitoring and interventions globally (Calero-Cáceres et al., 2024).

### 3.3 Technical considerations for genomic AMR surveillance

The reliability of genomic AMR surveillance depends on well-defined sequencing protocols, encompassing sample processing, sequencing methodology, and bioinformatics workflows (Figure 1). For isolate-based WGS, high-quality genomic DNA extraction, rigorous quality control, and standardized library preparation are crucial for accurate resistance detection (Sherry et al., 2023). Short-read sequencing platforms (e.g., Illumina) offer high accuracy for single nucleotide polymorphism (SNP) detection, ARG annotation, phylogenetic analysis, and genome-wide association studies (GWAS). Long-read technologies (e.g., Oxford Nanopore, PacBio) facilitate complete genome assemblies, precise plasmid reconstruction, and structural variation analysis (Bejaoui et al., 2025). Sequencing depth depends on study objectives: ≥100 × coverage is needed for precise SNP detection, plasmid tracking, and outbreak investigations, whereas 30–50 × coverage suffices for broader epidemiological surveillance and resistance gene identification.

For metagenomic surveillance, optimized DNA extraction methods are critical due to the complexity and variability of environmental samples, where resistance genes may be present at low abundances (Calero-Cáceres et al., 2024). Library preparation must minimize biases related to mixed microbial communities, and sequencing platform selection should balance resolution, accuracy, and cost-efficiency (Poulsen et al., 2022). Short reads offer affordable, high-throughput characterization of complex resistomes, whereas long reads enhance detection of MGEs, including plasmids and integrons. Sequencing depth must balance sensitivity for rare resistance genes with financial feasibility.

Regardless of approach, standardized bioinformatics pipelines are essential for consistent ARG annotation, strain typing, and comparative genomic analyses (Bogaerts et al., 2021). Integration with globally accessible AMR databases (e.g., NCBI, Pathogenwatch, ResFinder) will enhance data interoperability and facilitate real-time monitoring across sectors. Global harmonization of these technical elements is critical for robust AMR genomic surveillance, enabling early identification and response to emerging resistance threats (Argimón et al., 2021; Florensa et al., 2022).

## 4 Final considerations

Integration of whole-genome sequencing (WGS) and shotgun metagenomics is essential for a comprehensive AMR surveillance strategy, bridging high-resolution pathogen characterization with broader resistome analysis across diverse One Health ecosystems. While WGS enables precise strain tracking, outbreak investigations, and detailed genetic profiling of resistance, metagenomics provides critical insights into transmission dynamics, environmental reservoirs, and emergent resistance threats. Together, these approaches facilitate proactive rather than reactive surveillance, supporting timely interventions and evidence-based containment measures.

To fully harness genomic surveillance potential, global efforts must prioritize standardizing sequencing methodologies, harmonizing bioinformatics pipelines, and ensuring equitable access to genomic technologies, particularly in LMICs. A coordinated, cross-sectoral genomic surveillance framework integrating both WGS and metagenomics will enhance real-time monitoring, inform data-driven policy decisions, and support unified global responses to mitigate the rapidly evolving threat of antimicrobial resistance.

## Data Availability

The original contributions presented in the study are included in the article/supplementary material, further inquiries can be directed to the corresponding author.
